# Distribution and features of hematological malignancies in Eastern Morocco: a retrospective multicenter study over 5 years

**DOI:** 10.1186/s12885-016-2205-5

**Published:** 2016-02-25

**Authors:** Mounia Elidrissi Errahhali, Manal Elidrissi Errahhali, Redouane Boulouiz, Meryem Ouarzane, Mohammed Bellaoui

**Affiliations:** Medical Biology Unit, Faculty of Medicine and Pharmacy of Oujda, University Mohammed the First, Oujda, Morocco

**Keywords:** Hematological malignancies, Myeloid neoplasms, Lymphoid neoplasms, Morocco

## Abstract

**Background:**

Hematological malignancies (HM) are a public health problem. The pattern and distribution of diagnosed hematological cancers vary depending on age, sex, geography, and ethnicity suggesting the involvement of genetic and environmental factors for the development of these diseases. To our knowledge, there is no published report on HM in the case of Eastern Morocco. In this report we present for the first time the overall pattern of HM for this region.

**Methods:**

Retrospective descriptive study of patients diagnosed with HM between January 2008 and December 2012 in three centres in Eastern Morocco providing cancer diagnosis, treatment or palliative care services. The FAB (French-American-British) classification system has been taken into account in the analysis of myeloid and lymphoid neoplasms.

**Results:**

In this study, a total of 660 cases of HM were registered between January 2008 and December 2012. Overall, 6075 cases of cancers all sites combined were registered during this study period, indicating that HM account for around 10.9 % (660/6075) of all cancers recorded. Among the 660 registered cases of HM, 53 % were males and 47 % were females, with a male to female ratio of 1.1. Thus, overall, men are slightly more affected with HM than women. By contrast, a female predominance was observed in the case of Hodgkin’s lymphoma (HL), myeloproliferative neoplasms (MPN), acute myeloid leukemia (AML) and the myelodysplastic syndrome (MDS). HM occur at a relatively young age, with an overall median age at diagnosis of 54 years. Non-Hodgkin’s lymphoma (NHL) was the most common HM accounting for 29.7 % of all HM, followed by HL, MPN, multiple myelomas (MM), chronic lymphocytic leukemia (CLL), AML, MDS, acute lymphoblastic leukemia (ALL), and Waldenström macroglobulinemia (WM). The majority of HM cases have been observed among patients aged 60 years and over (40.4 % of HM). Among this age group, NHL was the most common HM. In adolescents, HL was the most frequent HM.

**Conclusions:**

This study provided for the first time the pattern and distribution of HM in Eastern Morocco. Our findings justify the need to establish a regional cancer registry as a first step in blood cancer control in Eastern Morocco.

**Electronic supplementary material:**

The online version of this article (doi:10.1186/s12885-016-2205-5) contains supplementary material, which is available to authorized users.

## Background

The HM are a group of cancers that arise from a malignant transformation of cells of the bone marrow or the lymphatic system [1]. Several classification systems have been developed over the past several years to subdivide the HM by relevant clinical and biological means. These classifications facilitate the recognition of HM and therefore refine our ability to diagnose and treat these cancers. Moreover, they enable the use of the same definition of a specific hematopoietic neoplasm, so that data comparison between studies is possible [2]. These classifications have been updated since the early 1970s, and in 2001 the World Health Organization (WHO) published a classification of tumours of the hematopoietic and lymphoid tissues. This 2001 WHO classification was the first worldwide consensus classification on hematological tumours and was based on multiple information, such as clinical, morphologic, biologic, immunophenotypic and genetic features [3, 4]. In 2008, the WHO published a new classification for hematopoietic and lymphoid neoplasms in conjunction with the Society for Hematopathology and the European Association of Hematopathology [3, 5]. This 4th edition of the WHO classification has refined the 2001 classification on the basis of new and cumulative experimental evidence [2, 5–11]. It includes new criteria for the recognition of some previously described neoplasms and information about new entities that have been defined mainly by genetic mutations that have been characterized only recently [5, 6, 11].

There are two major groups of HM according to their cell lineage: myeloid and lymphoid [7, 11]. Lymphoid neoplasms are a very varied group and the common subgroups are: NHL, HL, MM, WM, ALL, and CLL. Myeloid neoplasms are represented mainly by MPN, MDS and AML [5].

According to the most recent data, HM are estimated to represent about 6.5 % of all cancers worldwide in 2012 [12]. In the same year, NHL represented 2.7 % of all cancers and 2.4 % of all deaths from cancer worldwide. Leukemia accounted for 2.5 % of all cancers and 3.2 % of all deaths. MM represented 0.8 % of all cancers and 1.0 % of cancer deaths, while HL represented 0.5 % of all cancers and 0.5 % of cancer deaths [12]. The incidence of HM varies from one country to another. It varies with geography, age and ethnicity, suggesting the involvement of different etiological factors for these diseases.

In Morocco, there is no national database on hematological cancers, with the exception of some basic data found in the population-based cancer registries of Rabat and Casablanca [13–15]. In Eastern Morocco, there is no population-based cancer registry or published epidemiology report on HM. Therefore, in this study, we present for the first time the epidemiological aspects of HM in Eastern Morocco. Analysis of their distribution and their relative frequency compared to all cancers in this region is also conducted.

## Methods

Eastern Morocco is located in the north east of the Kingdom of Morocco, and is the third largest region of the Kingdom. According to the High Commission for Planning (HCP), Eastern Morocco had a population of over 2 million in 2013. The population is mainly urban (67 % vs. 33 % rural) and young, nearly 6 out of 10 people are under 30 years [16]. Our retrospective study was carried out in three centres: (1) Al-Farabi Regional Hospital (ARH) with its hematology and internal medicine units, which typically manage HM cases. (2) The Boussif Diagnostic Center (BDC), which also diagnoses HM cases. (3) Hassan II Regional Oncology Center (ROC), which normally manages all solid cancer cases. The study population consists of all patients diagnosed with cancer at the participating centres between January 2008 and December 2012. Among these, all cases of HM have been retrieved and reviewed carefully. For these cases, the data collected included details such as name, gender, age, place of residence, date of diagnosis, and type of HM. We have encountered some practical difficulties related to data collection since there is no methodic and effective system for data recording at the participating centres. For example, the same patient happens to be registered at the same center with slightly different names. So, before starting the analysis of the actual data, we proceeded to the elimination of duplicate cases. This step was necessary to avoid overestimating the actual case of diagnosed cancers.

The FAB (French-American-British) classification system has been taken into account in the analysis of myeloid and lymphoid neoplasms [17–19]. This classification was used instead of the 2008 WHO classification because of the lack of immunophenotypic, cytogenetic and molecular data necessary for the 2008 WHO classification. Data collection was performed on Excel. Statistical analysis was performed using SPSS software version 21.0. For the Chi-squared test, the results are considered significant when p (degree of significance) is less than 0.05, very significant when *p* < 0.01 and highly significant when *p* < 0.001.

In this retrospective study, obtaining informed consent was not possible. So, we were granted a waiver of consent by the Ethical Review Committee, and patient records/information was anonymized and de-identified prior to analysis. The study was approved by the Ethic Committee of the Faculty of Medicine and Pharmacy of Casablanca under the number 41/14. The authorization for personal data processing was obtained from the National Commission of control of Personal Data Protection under the number A-RS-280/2014.

## Results

In this study, 660 cases of hematological neoplasms were registered between January 2008 and December 2012 in the three participating centres. Overall, 6075 cases of cancers all sites combined were registered in these centres, indicating that HM account for around 10.9 % (660/6075) of all cancers recorded (Table 1). Lymphoid neoplasms were more common with 8.4 % of all cancers, and myeloid neoplasms accounted for 2.2 % of all cancers (Table 1). NHL was the most frequent HM (3.2 % of all cancer cases), with a median age of 55 years. HL was the second most common HM (1.7 %, median age 34 years), followed respectively by MPN (1.4 %, median age 53 years), MM (1.4 %, median age 63.5 years), CLL (0.6 %, median age 67 years), AML (0.5 %, median age 42.5 years), MDS (0.3 %, median age 62 years), ALL (0.3 %, median age 48 years), and finally WM (0.1 %, median age 46.5Years) (Table 1). The median age difference observed between the various types of HM was statistically significant (*p* < 0.05).

**Table 1:** Distribution pattern, median age at diagnosis and male to female ratio of hematological malignancies in Eastern Morocco

	All patients					Males		Females	
	N.	% of all cancers	% of HM	Median age	M/F	N.	% of HM	N.	% of HM
All HM	660	10.9	100	54	1.1	347	100	313	100
Lymphoid neoplasm	507	8.4	76.8	55	1.2	281	81.0	226	72.2
NHL	196	3.2	29.7	55	1.3	111	32.0	85	27.2
HL	105	1.7	15.9	34	0.9	50	14.4	55	17.6
MM	82	1.4	12.4	63.5	1.2	44	12.7	38	12.1
CLL	38	0.6	5.8	67	2.8	28	8.1	10	3.2
ALL	16	0.3	2.4	48	1.3	9	2.6	7	2.2
WM	4	0.1	0.6	46.5	^a^	0	0	4	1.3
Others^b^	66	1.1	10	-	-	39	11.2	27	8.6
Myeloid neoplasm	136	2.2	20.6	53	0.7	57	16.4	79	25.2
MPN	84	1.4	12.7	53	0.7	34	9.8	50	16.0
AML	32	0.5	4.9	42.5	0.9	15	4.3	17	5.4
MDS	20	0.3	3.0	62	0.7	8	2.3	12	3.8
Unspecified HM	17	0.3	2.6	-	-	9	2.6	8	2.6

*HM* hematological malignancies, *N* number of cases; % of all cancers: Percentage amongst all cancers; % of HM: percentage amongst hematological malignancies; ^a^: all patients were females; ^b^: Other lymphoid neoplasms; *M/F* male to female ratio; -: not applicable

HM all types combined were slightly more common in men than in women with a male to female ratio of 1.1 (Table 1). This slight male predominance was statistically significant (*p* < 0.05). In males, NHL was the most frequent HM with 32 % of all male hematological cancers, followed respectively by HL (14.4 %), MM (12.7 %), MPN (9.8 %), CLL (8.1 %), AML (4.3 %), ALL (2.6 %), and finally MDS (2.3 %) (Table 1). In females, NHL was also the most frequent HM with 27.2 % of all female hematological cancers followed respectively by HL (17.6 %), MPN (16 %), MM (12.1 %), AML (5.4 %), MDS (3.8 %), CLL (3.2 %), and finally ALL (2.2 %). Only 4 cases of WM were registered in our study and were all female cases (Table 1).

In our study, the overall median age at diagnosis for all HM combined was 54 years (Table 1). As with most cancers, the probability of being diagnosed with a HM increases markedly with age. However, unlike many other cancers, HM can be diagnosed at any age. Among the 660 registered cases of HM, the age at diagnosis was available for 651 cases. Of these, 51 cases were diagnosed in patients under 20 years of age, 119 cases in patients aged 20-39 years, 218 cases aged 40-59 years, and 263 cases were observed in patients aged 60 and over. The distribution of HM varies according to age group, and this difference was statistically significant (*p* < 0.05).

**Fig. 1:**
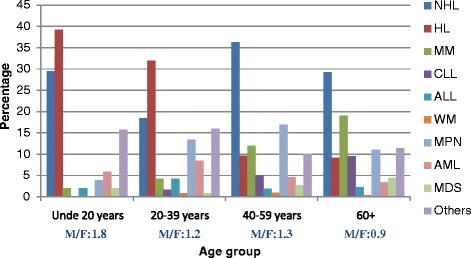
Distribution and male to female ratio (M/F) of hematological malignancies by age group in Eastern Morocco, 2008-2012

Indeed, in patients under 20 years of age, HL was the most common with 39.2 % of HM diagnosed in this age group, followed respectively by NHL (29.4 %), AML (5.9 %), MPN (3.9 %); and ALL, MDS and MM (equally represented with 2 %) (Fig. 1, Additional file 1). In this age group, there is a male predominance with a male to female ratio of 1.8 (Fig. 1).

In young adults aged 20-39 years, HL was also the most frequent with 31.9 % of HM diagnosed in this age group, followed respectively by NHL (18.5 %), MPN (13.5 %), AML (8.4 %), ALL and MM (equally represented with 4.2 %), CLL (1.7 %), MDS and WM (equally represented with 0.8 %) (Fig. 1, Additional file 1). In this age group, there is a slight male predominance with a male to female ratio of 1.2 (Fig. 1).

In adults aged 40-59 years, NHL was predominant with 36.2 % of HM diagnosed in this age group, followed respectively by MPN (17.0 %), MM (11.9 %), HL (9.6 %), CLL (5.0 %), AML (4.6 %), MDS (2.8 %), ALL (1.8 %) and WM (0.9 %) (Fig. 1, Additional file 1). In this age group, there is a male predominance with a male to female ratio of 1.3 (Fig. 1).

In older individuals over 60 years old, NHL was the most common with 29.3 % of HM, followed respectively by MM (19.0 %), MPN (11.0 %), CLL (9.5 %), HL (9.1 %), MDS (4.6 %), AML (3.4 %), ALL (2.3 %) and WM (0.4 %) (Fig. 1, Additional file 1). In this age group, there is a female predominance with male to female ratio of 0.9 (Fig. 1). The sex ratio difference between the various age groups was statistically significant at (*p* < 0.05).

**Fig. 2:**
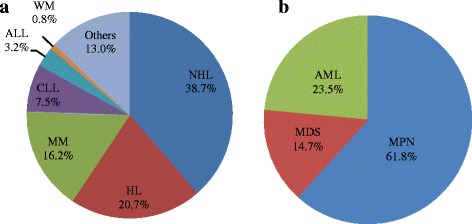
**a**. Distribution of NHL, HL, MM, CLL, ALL and WM amongst all lymphoid neoplasms (Others: Other lymphoid neoplasms). **b**. Distribution of MPN, AML and MDS amongst all myeloid neoplasms in Eastern Morocco, 2008-2012

**Fig. 3:**
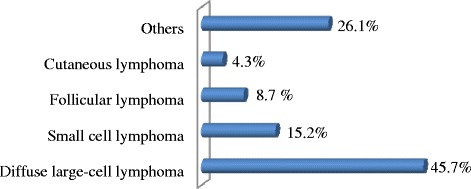
Distribution of NHL cases according to the histological type (Others: other histological types) in Eastern Morocco, 2008-2012

**Fig. 4:**
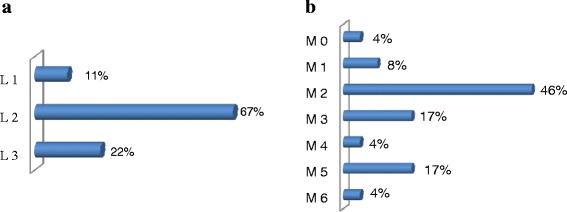
**a**. Distribution of ALL subtypes according to FAB classification. **b**. Distribution of AML subtypes according to FAB classification in Eastern Morocco, 2008-2012

In the group of lymphoid neoplasms, NHL was the most common disease with 38.7 % of all lymphoid neoplasms, followed respectively by HL (20.7 %), MM (16.2 %), CLL (7.5 %), ALL (3.2 %), and WM (0.8 %) (Fig. 2a). Among the 196 NHL cases recorded during the study period, only 46 cases had information on the histological type available. The analysis of these showed that the diffuse large cell lymphomas are the most common (45.7 %), followed respectively by diffuse small cell lymphomas (15.2 %), follicular lymphoma (8.7 %) and cutaneous lymphoma (4.3 %) (Fig. 3). Analysis of ALL showed that L2 was the predominant FAB subtype with 67 % of ALL, followed respectively by L3 (22 %) and L1 (11 %) (Fig. 4a). For myeloid neoplasms, MPN were the most frequent with 61.8 % of all myeloid neoplasms, followed by AML (23.5 %) and MDS (14.7 %) (Fig. 2b). Among FAB subtypes of AML, the commonest was M2 (46 %) followed by M3 and M5 (17 % each), M1 (8 %), and M0, M4 and M6 (4 % each) (Fig. 4b).

## Discussion

To our knowledge, this is the first epidemiological study on HM in Eastern Morocco. It was carried out using data collected from three different tertiary health care facilities available for management of cancer patients. It presents for the first time the relative frequency of several types of HM compared to all cancers in this region of Morocco. In this study, HM constituted 10.9 % of all cancers. This result is similar to those of Rabat and Casablanca [13, 15, 20]. In The United-States, HM accounted for 9,3 % of all cancers [21], which is similar to our data, but a much higher percentage was observed for hematological cancers in other studies: (18.05 % in Nigeria, 20 % in Iran and 24.8 % in Yemen) [22–25]. We found that lymphoid neoplasms are the most common, accounting for 76.8 % of all HM. This result is similar to those observed in cancer registries of Rabat, and Casablanca [13, 15]. Lymphoid neoplasms are also predominant in North America, Australia, Europe, Africa and Asia [26–30].

### Sex-ratio and age of HM in Eastern Morocco

#### *Sex-ratio of* HM

We found that overall men are slightly more affected with HM than women, with a male to female ratio of 1.1 (*p* < 0.05). Such a finding is similar to those reported in France and in the United Kingdom, where male to female ratio was 1.2 and 1.3, respectively [28, 30]. However, the sex ratio was much higher in Bangladesh and Senegal with a male to female ratio of 2.2 and 1.6, respectively [29, 31]. In the population of Rabat, males were also much more affected than females with HM [15, 20].

Although there is a slight male predominance among HM overall, the sex ratio varied between age groups. In our study, a strong male predominance was observed in the age group 0-19 years, with a male to female ratio of 1.8. A similar result was observed in the Casablanca registry for this age group, with a male to female ratio of 2.5 [13]. Several studies have shown that in developing countries HM in children often affect boys more than girls [32]. For the age groups 20-39, 40-59 and 60 years and over, the female to male ratio was 1.2, 1.3 and 0.9, respectively. This underlines the importance of the sex variable in the development of HM, as has been noted previously [33]. Further research is needed to understand the sex differences observed in Eastern Morocco.

#### *Median age at diagnosis of* HM

Our study revealed that HM in Eastern Morocco occur in adult population with an overall median age at diagnosis of 54 years (mean age 51.96 years). In Western countries, the HM usually affect older people. For example, in the United Kingdom, the median age at diagnosis was 70.6 years [28]. However, in Asia, HM usually affect the younger population. For example, in Bangladesh, the median age was 42 years [31]. Thus, the median age at diagnosis of HM in Eastern Morocco is younger than that found in Western countries, but it is higher than that in Asia. It is important to note that the real median age in Eastern Morocco could be even lower, given the under-representation of children in this study because childhood cancer cases are normally treated in other services. Several factors may be involved in the occurrence of HM at relatively young ages such as shorter life expectancy, younger population, genetic and environmental factors. Further studies are needed to confirm the role of these factors in the young age of HM in Eastern Morocco.

#### HM in patients aged over 60 years

In our study, the majority of hematological cancer cases have been observed among patients aged 60 years and over (40.4 % of MH). This result is predictable since cancer is triggered after several genetic mutations that accumulate with age [34, 35]. In this age group, there was a female predominance with a male to female ratio of 0.9. The greater longevity of women might be partly responsible for this female predominance among elderly patients. In this age group, NHL was the most common (29.3 % of HM), followed respectively by MM (19.0 %), MPN (11.0 %), CLL (9.5 %), HL (9.1 %), MDS (4.6 %), AML (3.4 %), ALL (2.3 %) and WM (0.4 %). The higher prevalence of HM in this age group justifies the need to develop effective programs to improve the management of hematological malignant diseases in the elderly, especially when they are associated with comorbidity factors [36, 37].

#### HM in adolescents

In adolescents (15-19 years age group) lymphomas were predominant and accounting for 64.4 % of HM (HL-33.3 %, NHL-31.1 %). Lymphomas were also the most common cancers among adolescents in Europe, North America, Middle East, Algeria and several African countries [38–42]. However, in Asia and Latin America, leukemias were the most frequent cancers in adolescents [31, 42, 43]. The distribution of lymphomas in our study is comparable to that in Europe, North America and Israel where HL is more frequent than NHL [38, 42]. However, in Algeria and in most African countries, HL is less common than NHL [38–42]. Infection with Epstein-Barr virus (EBV) has been associated with most cases of NHL or HL [44–47]. Other risk factors have been associated with HM in children such as diet, paternal smoking, genetic and environmental factors, but the results are inconclusive [48–50]. Further studies are needed to understand the reasons for this distribution of HM in adolescents in Eastern Morocco.

### Features of lymphoid neoplasms in Eastern Morocco

#### Non-Hodgkin’s lymphoma

In this study, NHL was the most frequent common HM. NHL was also the most frequent HM in cancer registries of Rabat, Casablanca and other African countries [13, 15, 20, 23, 51, 52]. In developed countries, NHL is also the most prevalent type of HM, with the highest incidence in the United States, Australia, New Zealand and Europe [53]. In Asian countries like Japan, Korea and Yemen, NHL is also the commonest cancer among HM. However, in other countries including India and Bangladesh, leukemia cases were the most frequent HM [12, 31, 43, 53, 54].

**Fig. 5:**
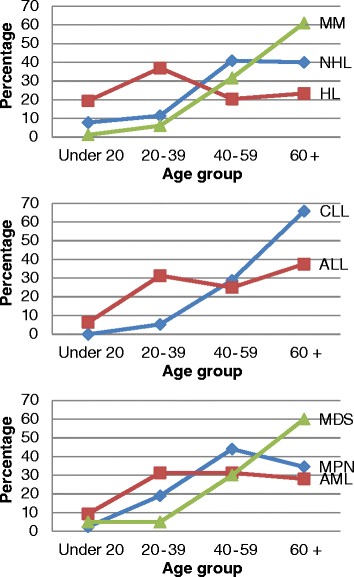
Age-group specific distribution of hematological malignancies in Eastern Morocco, 2008-2012

NHL was more common in men than in women with a male to female ratio of 1.3. Such a finding is similar to that reported in Casablanca, where male to female ratio was 1.53 [13, 55]. However, the sex ratio was much higher in Rabat with a male to female ratio of 2.25 [20]. The frequency of NHL increases with advancing age, but affected equally adults and elderly (Fig. 5). We observed that 7.8 % of all NHL were under 20 years, 37.9 % were below age 50 and 39.9 % were over 60 years (Fig. 5, Additional file 1). This age distribution of NHL is similar to that observed in Casablanca where patients under 20 accounted for 8 % of all NHL cases, patients younger than 50 years accounted for 38 %, while those over 60 years accounted for 42.1 % [13, 55]. In our study we found that diffuse large cell lymphoma was the most common form of NHL (45.7 %), which is consistent with the Rabat and Casablanca studies, which also reported a predominance of diffuse large cell lymphoma with 46.9 % and 50.1 %, respectively [20, 55]. Diffuse small cell lymphoma constituted 15.2 % of NHL, which is lower compared to Casablanca (19.2 %) [55]. Follicular lymphoma constituted 8.7 %, which is higher than the figure suggested by the Rabat study (6.2 %) [20]. Cutaneous lymphoma constituted 4.3 % of NHL, which is comparable to that in Casablanca (3.3 %), and Rabat (6.2 %) [20, 55]. Further extensive study is necessary to understand this difference in the distribution of the various form of NHL between Eastern Morocco and other regions of Morocco.

#### Hodgkin’s lymphoma

In this study, HL was the second most common blood cancer after NHL, accounting for 15.9 % of all HM. This frequency is lower than that observed in the cancer registry of Casablanca (between 17 % and 21.4 %), but it is higher than those observed in Rabat (10.4 %), United States (5.87 %), and France (5 %) [20, 21, 30, 55, 56]. A lower frequency was observed in other studies in Asian countries including China, Japan, India, Korea and Bangladesh [31, 57–60]. In our study, HL occurred in people of all ages with a peak in young adults (36.9 % of all patients with LH occurred in the age group 20-39 years) (Fig. 5, Additional file 1). In developed countries, the age distribution of HL is bimodal, the first peak occurs in young adults (20-34 years) and the second peak in the older age group (55-74 years). In contrast, in developing countries, the first peak occurs in children (under 20), probably because of an earlier infection with the Epstein-Barr virus in children living in those countries [61, 62]. Therefore, the age distribution of HL in Eastern Morocco resembles that of developed countries with a peak in young adults.

In our study, we found that women are slightly more affected with HL than men, with a male to female ratio of 0.9. Such a finding is far from that reported in other studies, where HL is much more frequent in men than in women with a male to female ratio between 1.5 and 2 [33, 63, 64]. In Casablanca and Rabat, it was reported that HL affects males and females, with an equal ratio [13, 20]. Additional studies are needed to better understand these sex ratio differences.

#### Multiple Myeloma

MM is the fourth most common blood cancer in Eastern Morocco, accounting for 12.4 % of all HM. This frequency of MM is comparable to those in the United States (13 %), France (13.7 %) and UK (10.5 %) [28, 30, 65]. We found that men are slightly more affected with MM than women, with a male to female ratio of 1.2, which is consistent with other studies [33, 65, 66]. The frequency of MM increases with advancing age: 1.2 % MM occurred in children under 20 years, 6.1 % in young adults, 31.7 % in adults and 61 % in patients aged 60 years and over (Fig. 5, Additional file 1). The overall median age at diagnosis was 63.5 years, which is significantly higher as compared to Asian countries where the median age is around 55 years [31, 67], but it is lower as compared to Western countries, where the median age is between 65 and 70 years [68].

#### Chronic lymphoid leukemia

In this study, CLL constituted 5.8 % of all HM. This frequency is higher as compared to those observed in Asian countries [31, 69, 70], and lower as compared to what has been reported in Western countries [27, 28, 30, 71]. In the US, the incidence of CLL is lower among Asians and African-Americans as compared to Caucasians [72]. Therefore, multiple factors may play an important role in the development of CLL, including genetic and environmental factors [71–73].

We found that the median age at diagnosis for CLL was 67 years. This result is intermediate between the results found in Western countries (median age of 70-72 years) and Asia (median age of 59-60 years) [28, 30, 31, 70, 74]. We found that the frequency of CLL increases with advancing age. Indeed, no cases of CLL have been observed in children under 20 years, 5.3 % of CLL occurred in young adults, 28.9 % in adults and 65.8 % in patients aged 60 years and over (Fig. 5, Additional file 1). There is a male predominance among CLL, with a male to female ratio of 2.8, which is in accordance with studies reported from India (sex ratio of 3), Bangladesh (sex ratio of 2.9), Ethiopia (sex ratio of 3.6), and Western countries (sex ratio of 1.5–2) [30, 31, 70, 71, 75].

#### Acute lymphocytic leukemia

ALL constituted 2.4 % of all HM. This frequency is similar to those reported from France (2.7 %), UK (2 %) and United States (3.84 %) [21, 28, 30], but lower as compared to those observed in Asian countries such as Yemen (4 %) and Bangladesh (14.1 %) [22, 31]. ALL is generally most common in childhood and its age incidence curve is bimodal, with a peak in childhood and another peak in old age [30, 63, 76]. Our study showed a different profile, with the first peak occurring in the 20-39 years age group, and the second peak in old age (Fig. 5, Additional file 1). Only 6.3 % of ALL was observed in children aged under 20 years old, and thus the childhood peak was absent. This difference could be explained by the under-representation of children in our study, since children HM are usually treated in other services. We also found that L2 was the predominant FAB subtype (67 %), followed by L3 (22 %) and L1 (11 %), which is consistent with the age distribution of ALL in our study since L2 is more often observed in adults and most childhood cases are of subtype L1 [77].

### Features of myeloid neoplasms in Eastern Morocco

#### Myeloproliferative neoplasms

In our study, MPN constituted 12.7 % of all HM. This frequency is comparable to that reported in France (11 %), and the UK (9 %) [28, 30]. We found that MPN were more common in women than in men, with a male to female ratio of 0.7. A female predominance was also observed in the United Kingdom (sex ratio of 0.8) and France (sex ratio of 0.94) [28, 30]. We also found that MPN were more common in adults and elderly but also affected younger patients (Fig. 5, Additional file 1). The mean age of diagnosis was 52.8 years, which is lower than the one observed in France (65) [30]. The younger age phenomenon of HM was discussed earlier in this discussion.

#### Acute myeloid leukemia

AML constituted 4.9 % of all HM. This frequency is comparable to that in Yemen (4 %), but it is lower than those reported in France (7 %), UK (6.68 %), United States (12 %) and Bangladesh (28.3 %) [21, 22, 28, 30, 31]. We found that AML is more common in women than in men with a male to female ratio of 0.9 which is different from what was found in other studies [28, 30, 31, 76, 78]. Our study showed that AML affected equally young adults, adults and elderly (Fig. 5, Additional file 1). However, in Western countries, AML generally affects older people with a median age of 65-67 years [71, 79]. In France, for example, 60.6 % of AML was observed in people aged 60 years and over [30]. A different picture is observed in Asia, where AML affects young adults with a median age at diagnosis of 30 years in India and 35 in Bangladesh [31, 80]. In our study, the distribution of AML according to the FAB classification, has revealed that M2 was the predominant FAB subtype (46 %), followed by M3 and M5 (17 %), M1 (8 %), M0, M4 and M6 (4 %). This distribution was comparable to that reported in China [81]. The predominance of M2 was also observed in Iran, India, Germany, USA and Singapore [82–86].

#### Myelodysplastic syndrome

MDS constituted 3 % of all HM, which is lower to what was reported in Bangladesh (4.5 %), UK (6 %), and France (12.8 %) [28, 30, 31]. We found that MDS affected elderly with a median age of 62 years, and 60 % of the MDS patients aged over 60 years (Fig. 5, Additional file 1). This result is similar to that reported in Western countries where MDS typically affects elderly people with a median age at diagnosis between 60 and 70 years [28, 30]. However, in Asian countries, MDS typically affects people who are much younger [31, 43, 80]. The median age was 46.1 years in India and 57 years in Bangladesh [31, 87]. We found that MDS is more common in women than in men with a male to female ratio of 0.7, which is different from what was found in other studies. For example, the sex ratio was 1.38 in France, 2 in UK, and 1.9 in Bangladesh [28, 30, 31].

## Conclusion

This study provided for the first time the pattern and distribution of HM in Eastern Morocco. HM occur at a relatively young age, with an overall median age at diagnosis of 54 years. Overall, men are slightly more affected with HM than women, with a male to female ratio of 1.1. NHL was the most common HM accounting for 29.7 % of all HM, followed by HL, NMP, MM, CLL, AML, MDS, ALL, and WM. The majority of HM cases have been observed among patients aged 60 years and over (40.4 % of MH). In adolescents, HL was the most frequent HM.

Our findings justify the need to establish a regional cancer registry as a first step in blood cancer control in Eastern Morocco. Further studies are necessary to better understand and develop a more effective program aiming at controlling and preventing the spread of blood cancers in Eastern Morocco.

## Additional file

Additional file 1: Includes two tables: (Table S1) showing the distribution and male to female ratio of hematological malignancies by age group in Eastern Morocco for the study period, and (Table S2) showing the Age-group specific distribution of hematological malignancies in Eastern Morocco, 2008-2012. (PDF 32 kb)

## Abbreviations

ALL: Acute lymphoblastic leukemia
AML: Acute myeloid leukemia
CLL: Chronic lymphocytic leukemia
HL: Hodgkin’s lymphoma
HM: Hematological malignancies or Hematological malignancy
MDS: Myelodysplastic syndromes
MM: Multiple myeloma
MPN: myeloprofilerative neoplasms
NHL: Non-Hodgkin’s lymphoma
WM: Waldenström macroglobulinemia
