# Supplementary material for: Distribution and features of hematological malignancies in Eastern Morocco: a retrospective multicenter study over 5 years
Source: BMC Cancer. 2016 Feb 25;16:159. doi: 10.1186/s12885-016-2205-5 (PMC4768409; doi:10.1186/s12885-016-2205-5)
Supplement: Additional file 1: — Includes two tables: (Table S1) showing the distribution and male to female ratio of hematological malignancies by age group in Eastern Morocco for the study period, and (Table S2) showing the Age-group specific distribution of hematological malignancies in Eastern Morocco, 2008-2012. (PDF 32 kb) [file 12885_2016_2205_MOESM1_ESM.pdf]

Table S1: Distribution and male to female ratio (M/F) of hematological malignancies by age group in Eastern Morocco, 2008-2012

**Age group (Year)**	**MPN** (n) %	**AML** (n) %	**MDS** (n) %	**NHL** (n) %	**HL** (n) %	**MM** (n) %	**CLL** (n) %	**ALL** (n) %	**WM** (n) %	**Others** (n) %	**Total (n) %**	**H/F**
0-14	(0) 0	(0) 0	(0) 0	(1) 16.7	(5) 83.3	(0) 0	(0) 0	(0) 0	(0) 0	(0) 0	(6) 100	
15-19	(2) 4.4	(3) 6.7	(1) 2.2	(14) 31.1	(15) 33.3	(1) 2.2	(0) 0	(1) 2.2	(0) 0	(8) 17.8	(45) 100	
**Under 20**	(2) 3.9	(3) 5.9	(1) 2	(15) 29.4	(20) 39.2	(1) 2	(0) 0	(1) 2	(0) 0	(8) 15.7	(51) 100	1.8
20-29	(8) 12.7	(6) 9.5	(0) 0	(12) 19	(23) 36.5	(1) 1.6	(0) 0	(2) 3.2	(1) 1.6	(10) 15.9	(63) 100	
30-39	(8) 14.3	(4) 7.1	(1) 1.8	(10) 17.9	(15) 26.8	(4) 7.1	(2) 3.6	(3) 5.4	(0) 0	(9) 16.1	(56) 100	
**20- 39**	(16) 13.4	(10) 8.4	(1) 0.8	(22) 18.5	38) 31.9	(5) 4.2	(2) 1.7	(5) 4.2	(1) 0.8	(19) 16	(119) 100	1.2
40-49	(18) 17.8	(6) 5.9	(0) 0	(36) 35.6	(13) 12.9	(10) 9.9	(4) 4	(4) 4	(2) 2	(8) 7,9	(101) 100	
50-59	(19) 16.2	(4) 3.4	(6) 5.1	(43) 36.8	(8) 6.8	(16) 13.7	(7) 6	(0) 0	(0) 0	(14) 12	(117) 100	
**40-59**	(37) 17.0	(10) 4.6	(6) 2.8	(79) 36.2	(21) 9.6	(26) 11.9	(11) 5	(4) 1.8	(2) 0.9	(22) 10.1	(218) 100	1.3
60-69	(14) 12	(4) 3.4	(4) 3.4	(33) 28.2	(13) 11.1	(25) 21.4	(8) 6.8	(2) 1.7	(0) 0	(14) 12	(117) 100	
70+	(15) 10.3	(5) 3.4	(8) 5.5	(44) 30.1	(11) 7.5	(25) 17.1	(17)11.6	(4) 2.7	(1) 0.7	(16) 11	(146) 100	
**60 +**	(29) 11	(9) 3.4	(12) 4.6	(77) 29.3	(24) 9.1	(50) 19	(25) 9.5	(6) 2.3	(1) 0.4	(30) 11.4	(263) 100	0.9

Among the 660 registered cases of hematological malignancies, the age at diagnosis was available for only 651 cases (the age was missing for 3 cases NHL, 2 cases HL and 4 cases in others category); others: other lymphoid neoplasms and unspecified hematological malignancies; M/F: male to female ratio

Table S2: Age-group specific distribution of hematological malignancies in Eastern Morocco, 2008-2012

**Age group (Year)**	**MPN** (n) %	**AML** (n) %	**MDS** (n) %	**NHL** (n) %	**HL** (n) %	**MM** (n) %	**CLL** (n) %	**ALL** (n) %	**WM** (n) %	**Others** (n) %
0-14	(0) 0	(0) 0	(0) 0	(1) 0.5	(5) 4.9	(0) 0	(0) 0	(0) 0	(0) 0	(0) 0
15-19	(2) 2.4	(3) 9.4	(1) 5	(14) 7.3	(15) 14.6	(1) 1.2	(0) 0	(1) 6.3	(0) 0	(8) 10.1
**Under 20**	(2) 2.4	(3) 9.4	(1) 5	(15) 7.8	(20) 19.4	(1) 1.2	(0) 0	(1) 6.3	(0) 0	(8) 10.1
20-29	(8) 9.5	(6) 18.8	(0) 0	(12) 6.2	(23) 22.3	(1) 1.2	(0) 0	(2) 12.5	(1) 25	(10) 12.7
30-39	(8) 9.5	(4) 12.5	(1) 5	(10) 5.2	(15) 14.6	(4) 4.9	(2) 5.3	(3) 18.8	(0) 0	(9) 11.4
**20- 39**	(16) 19	(10) 31.3	(1) 5	(22) 11.4	(38) 36.9	(5) 6.1	(2) 5.3	(5) 31.3	(1) 25	(19) 24.1
40-49	(18) 21.4	(6) 18.8	(0) 0	(36) 18.7	(13) 12.6	(10) 12.2	(4) 10.53	(4) 25	(2) 50	(8) 10.1
50-59	(19) 22.6	(4) 12.5	(6) 30	(43) 22.3	(8) 7.8	(16) 19.5	(7) 18.4	(0) 0	(0) 0	(14) 17.7
**40-59**	(37) 44	(10) 31.3	(6) 30	(79) 40.9	(21) 20.4	(26) 31.7	(11) 28.9	(4) 25	(2) 50	(22) 27.8
60-69	(14) 16.7	(4) 12.5	(4) 20	(33) 17.1	(13) 12.6	(25) 30.5	(8) 21.1	(2) 12.5	(0) 0	(14) 17.7
70+	(15) 17.9	(5) 15.6	(8) 40	(44) 22.8	(11) 10.7	(25) 30.5	(17) 44.7	(4) 25	(1) 25	(16) 20.3
**60 +**	(29) 34.5	(9) 28.1	(12) 60	(77) 39.9	(24) 23.3	(50) 61.0	(25) 65.8	(6) 37.5	(1) 25	(30) 38
**Total**	(84) 100	(32) 100	(20) 100	(193) 100	(103) 100	(82) 100	(38) 100	(16) 100	(4) 100	79 (100)

Among the 660 registered cases of hematological malignancies, the age at diagnosis was available for only 651 cases (the age was missing for 3 cases NHL, 2 cases HL and 4 cases in others category); others: other lymphoid neoplasms and unspecified hematological malignancies
